# In inpatients with cirrhosis opioid use is common and associated with length of stay and persistent use post-discharge

**DOI:** 10.1371/journal.pone.0229497

**Published:** 2020-02-26

**Authors:** Andrew M. Moon, Yue Jiang, Shari S. Rogal, Jasper Becker, A. Sidney Barritt

**Affiliations:** 1 Division of Gastroenterology and Hepatology, University of North Carolina at Chapel Hill, Chapel Hill, NC, United States of America; 2 Department of Statistical Science, Duke University, Durham, NC, United States of America; 3 Division of Gastroenterology, Hepatology, and Nutrition, University of Pittsburgh, Pittsburgh, PA, United States of America; 4 North Carolina Translational and Clinical Sciences Institute (NC TraCS), University of North Carolina School of Medicine, Chapel Hill, NC, United States of America; Azienda Ospedaliero Universitaria Careggi, ITALY

## Abstract

**Background:**

Previous studies have demonstrated that opioids are often prescribed and associated with complications in outpatients with cirrhosis. Less is known about opioids among hospitalized patients with cirrhosis. We aimed to describe the patterns and complications of opioid use among inpatients with cirrhosis.

**Methods:**

This retrospective cohort study included adult patients with cirrhosis admitted to a single hospital system from 4/4/2014 to 9/30/2015. We excluded hospitalizations with a surgery, invasive procedure, or palliative care/hospice consult in order to understand opioid use that may be avoidable. We determined the frequency, dosage, and type of opioids given during hospitalization. Using bivariable and multivariable analyses, we assessed length of stay, intensive care unit transfer, and in-hospital mortality by opioid use.

**Results:**

Of 217 inpatients with cirrhosis, 118 (54.4%) received opioids during hospitalization, including 41.7% of patients without prior outpatient opioid prescriptions. Benzodiazepines or hypnotic sleep aids were given to 28.8% of opioid recipients. In the multivariable model, younger age and outpatient opioid prescription were associated with inpatient opioids. Hospitalization was longer among opioid recipients (median 3.9 vs 3.0 days, p = 0.002) and this difference remained after adjusting for age, cirrhosis severity, and medical comorbidities. There was no difference in intensive care unit transfers and no deaths occurred. At discharge, 22 patients were newly started on opioids of whom 10 (45.5%) had opioid prescriptions at 90 days post-discharge.

**Conclusion:**

In non-surgical inpatients with cirrhosis, opioid prescribing was common and associated with prolonged length of stay. A high proportion of patients newly discharged with opioid prescriptions had ongoing prescriptions at 90 days post-discharge.

## Introduction

Pain is common among patients with cirrhosis and analgesia for these patients can be difficult [1–3]. Given the presumed limitations of using high-dose acetaminophen and nonsteroidal anti-inflammatory drugs (NSAIDs), short-acting opioids are commonly used in patients with cirrhosis [2, 4]. The prevalence and potential complications of opioid use among those with cirrhosis remain unclear, particularly for hospitalized patients.

Previous studies have examined opioid prescriptions among patients with cirrhosis in the outpatient and hospital discharge settings [1, 4–6]. These studies identified patient characteristics associated with opioid prescription including sleep disturbance, psychiatric comorbidities, and past substance use. In these populations opioids were associated with increased overall health care utilization and hospital readmissions [5, 6].

The patterns of opioid prescribing among hospitalized patients with cirrhosis are relatively understudied. Opioids likely increase the risk of complications in patients with cirrhosis [5, 7, 8], especially when used in combination with benzodiazepines [9, 10]. Furthermore, inpatient opioids may lead to chronic use in some patients. Of all opioid naïve patients admitted to the hospital, 15–25% receive a new prescription for opioids, 43% of whom subsequently fill an opioid prescription 90 days post-discharge [11, 12]. This is concerning given opioids’ demonstrated harms and lacking efficacy data for chronic pain [13–16].

We therefore aimed to describe patterns of inpatient opioid prescribing among patients with cirrhosis at a single tertiary medical center and assess patient characteristics associated with inpatient opioid prescribing. We additionally evaluated the association between opioid prescriptions and length of stay, transfer to the intensive care unit (ICU), inpatient mortality, and 30-day readmission. Lastly, we assessed the prevalence and indications for opioid prescriptions at discharge and, for new opioid starts, determined whether patients remained on opioids at 30 days and 90 days post-discharge.

## Materials and methods

### Study design

This was a retrospective cohort study within the University of North Carolina (UNC) hospital system. All data were obtained by extraction through the Carolina Data Warehouse for Health (CDW-H) and subsequent review of the electronic medical record. The CDW-H is a central data repository containing administrative, research, and clinical data from the UNC Health Care System from mid-2004 to present. This project was approved by the UNC Institutional Review Board (IRB).

### Patient selection

We included all patients ≥18 years old admitted to UNC hospitals from April 4, 2014 to September 30, 2015 with ≥2 pre-admission International Classification of Diseases, 9th revision, clinical modification (ICD-9-CM) codes for cirrhosis or its complications (Table 1). This definition has been used and validated in previous publications [17–19]. The diagnosis of cirrhosis was verified in all patients by individual chart review. We excluded patients with prior liver transplant unless they developed recurrent cirrhosis. The start date of April 4, 2014 was chosen because this is when UNC hospitals began use of the Epic electronic medical record, which allowed complete capture of inpatient opioid use. The end date of September 30, 2015 was when ICD-9 was replaced with ICD-10 coding, which lacks the rigorous validation for definitions of cirrhosis and patient comorbidities.

**Table 1:** ICD-9-CM coding definitions.

	ICD-9 codes used
Cirrhosis	Two of the following: 571.2, 571.5, 456.0–456.21, 567.23, 572.2, 572.4
Esophageal varices with bleeding	456.0, 456.20
Spontaneous bacterial peritonitis	567.23
Hepatic encephalopathy	572.2
Hepatorenal syndrome	572.4
Hepatocellular carcinoma	155.00
Charlson Comorbidity Score	410–410.9 (myocardial infarction, Charlson score 1); 428–428.9 (congestive heart failure, Charlson score 1); 433.9, 441–441.9, 785.4, V43.4 (peripheral vascular disease, Charlson score 1); 430–438 (Cerebrovascular Disease, Charlson score 1); 290–290.9 (Dementia, Charlson score 1); 490–496, 500–505, 506.4 (Chronic Pulmonary Disease, Charlson score 1); 710.0, 710.1, 710.4, 714.0–714.2, 714.81, 725 (Rheumatologic Disease, Charlson score 1); 531–534.9 (Peptic Ulcer Disease, Charlson score 1); 571.2, 571.5, 571.6, 571.4–571.49 (Mild Liver Disease, Charlson score 1); 250–250.3, 250.7 (Diabetes, Charlson score 1); 250.4–250.6 (Diabetes with Chronic Complications, Charlson score 2); 344.1, 342–342.9 (Hemiplegia or Paraplegia, Charlson score 2); 582–582.9, 583–583.7, 585, 586, 588–588.9 (Renal Disease, Charlson score 2); 572.2–572.8 (Moderate or Severe Liver Disease, Charlson score 3); 042–044.9 (AIDS, Charlson score 6)
ESRD/hemodialysis	V45.1, V56.0, V56.1, CPT 39.95
Depression	296.20–25, 296.30–35, 300.4, 311
Anxiety	300.00–300.02
Substance abuse/dependence	305.00–305.95, 304.40–304.93

ICD-9-CM: International Classification of Diseases, 9th revision, clinical modification; AIDS: acquired immune deficiency syndrome; ESRD: end stage renal disease; CPT: Current Procedural Terminology

By chart review, we identified and excluded admissions with a recent (within 7 days of admission or during hospitalization) surgery or invasive procedure (e.g. chest tube, transcatheter arterial chemoembolization, transjugular intrahepatic portosystemic shunt). Minimally invasive procedures such as paracentesis and upper endoscopy were not considered invasive procedures in this definition. We also excluded hospitalizations in which a palliative care consult for pain management or comfort care/hospice referral occurred, given that the balance of opioids’ benefits and risks may be different for this population. We only considered a patient’s first admission.

### Patient characteristics

We extracted primary admission diagnosis and patient demographics including sex, race, and age. We calculated body mass index (BMI) at the time of admission as weight in kilograms divided by height in meters squared. We determined each patient’s admission team and limited our analysis to patients admitted to the internal medicine or family medicine inpatient services. This was done to minimize the inclusion of patients with painful surgical issues who did not receive surgery due to cirrhosis or other medical comorbidities. We identified outpatient prescriptions for opioids at the time of admission by individual chart review. We determined substance abuse/dependence, depression and anxiety based on the presence of ICD-9 codes before the date of admission. We calculated the Charlson Comorbidity Index (CCI) for each patient [20].

We determined etiology of liver disease by individual review of labs, imaging and clinical notes and categorized etiologies as hepatitis C virus (HCV), alcohol-associated, HCV/alcohol, non-alcoholic fatty liver disease (NAFLD), hepatitis B virus (HBV), and other. We assessed severity of liver disease via the Model for End Stage Liver Disease (MELD) score, MELD-Na score, and the presence of pre-admission decompensation events [21]. We extracted admission laboratory values and performed chart review to determine if patients were on hemodialysis within the prior week. For cirrhosis complications, we required at least two preadmission ICD-9 codes for esophageal varices, spontaneous bacterial peritonitis, hepatic encephalopathy, hepatorenal syndrome, or hepatocellular carcinoma (HCC) (Table 1). These definitions of cirrhosis complications have been previously validated and used [18, 22, 23].

### Assessment of opioid use and associated complications

Among patients who received opioids, we extracted data from the CDW-H to determine cumulative opioid doses from the time of first contact in the emergency department until ICU transfer, discharge, or death. We calculated the morphine milligram equivalent (MME) using standard tables [13, 24]. We excluded all opioids given for sedation (e.g., intravenous fentanyl) and patients who exclusively received intravenous fentanyl were considered to not have received opioids. We determined the indication for inpatient opioid prescriptions by reviewing the electronic medical record. We assessed whether patients were discharged with prescriptions for opioid medications and reviewed charts to assess the reasons for opioid prescriptions at discharge. For new opioid starts, we assessed if patients had a subsequent opioid prescription within a month of discharge and/or an active prescription at 90 days post-discharge. We also determined reasons for opioid discontinuations among patients with pre-admission opioid prescriptions.

We ascertained the in-hospital use of non-opioid analgesics including NSAIDs, acetaminophen, gabapentinoids (gabapentin/pregabalin), tricyclic antidepressants (TCAs), serotonin and norepinephrine reuptake inhibitors (SNRIs), muscle relaxants, and topical analgesics. Lastly, we determined if patients received benzodiazepines or hypnotic sleep aids during hospitalization.

We determined the total length of stay, ICU transfer, in-hospital mortality, and 30-day readmission using the CDW-H and confirmed these outcomes via individual chart review.

### Statistical analysis

Statistical analyses were carried out using R version 3.4.2 (Vienna, Austria).[25] We calculated patients’ total MME during hospitalization and determined median and interquartile range (IQR) for daily MME among opioid recipients. We determined the number of days of opioid use by counting the number of unique hospital days patients received opioids. We classified opioid use as a dichotomous variable (any/none) and presented patients’ daily opioid use (by MME) in a histogram, stratified by outpatient opioid prescription. We performed bivariable analyses to assess patient characteristics associated with inpatient receipt of opioids, using t-tests, χ^2^ tests, and Fisher’s exact tests where appropriate. To evaluate variables associated with inpatient prescription of opioids, a multivariable logistic regression model was created and included patient demographics, MELD, CCI, depression, and pre-admission outpatient opioid prescription. We used logistic regression to assess variables associated with ICU transfer and in-hospital mortality, including inpatient opioids, age, MELD, and CCI. Lastly, multivariable Poisson regression with robust standard errors was performed to assess variables associated with length of stay. For multivariable models, we included covariates that were deemed to be potential confounders in the relationship between opioid use and relevant outcomes. These variables were chosen *a priori*. P-values <0.05 (two-sided) were considered statistically significant in all analyses.

## Results

### Overall patient and hospitalization characteristics

**Fig 1:**
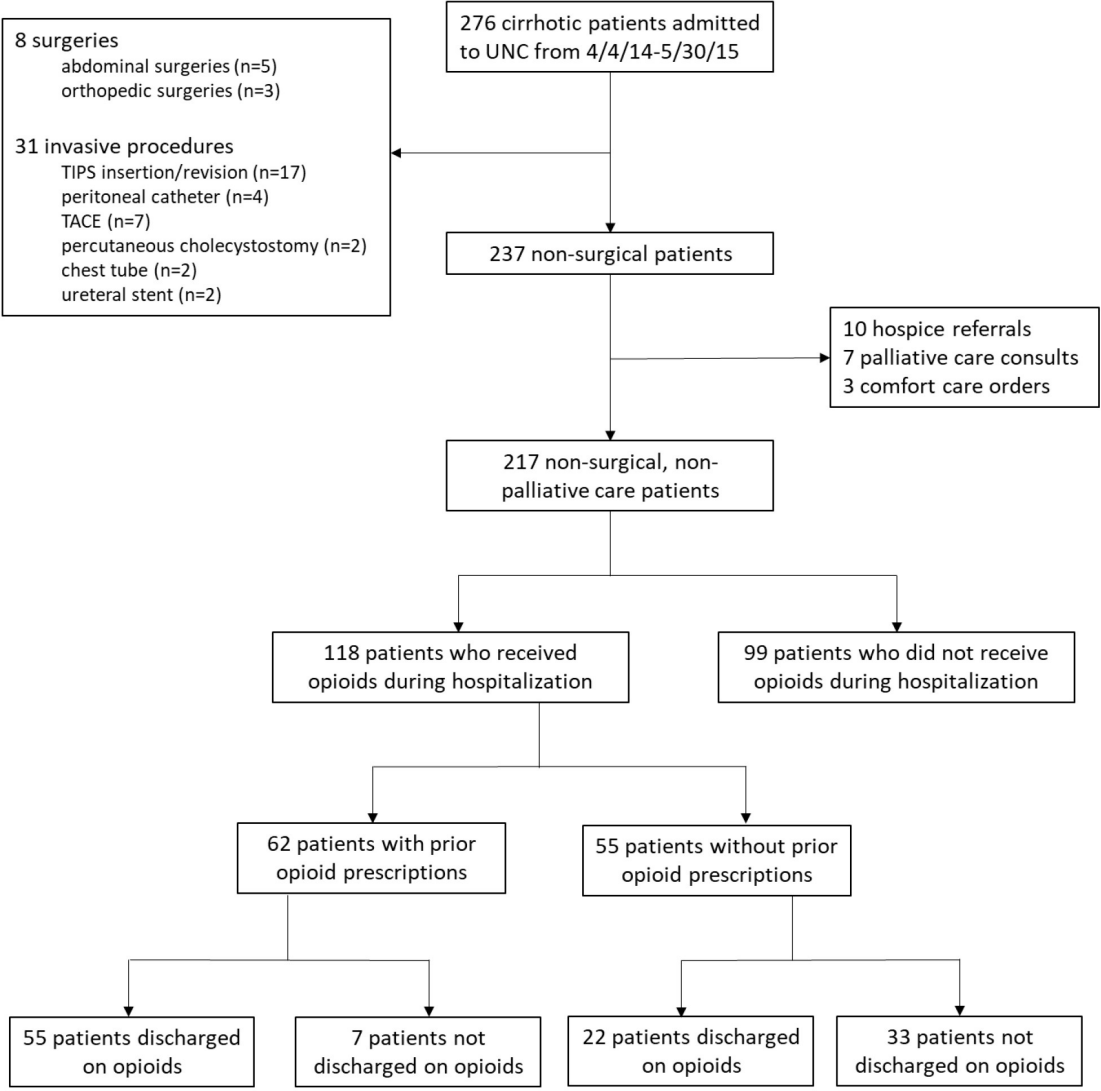
Flow diagram of inpatient cohort. Flow diagram demonstrating the number of patients with cirrhosis admitted to the University of North Carolina (UNC) hospitals during the study period and the numbers included in the analysis after excluding recent surgeries or invasive procedures or hospice/palliative care/comfort care consultations. Among those included in the final cohort, the proportion receiving opioids stratified by outpatient opioid prescription is shown. Lastly, the number of patients discharged on opioids is demonstrated.

After excluding patients with recent surgeries/invasive procedures or palliative care/hospice referrals, we identified 217 inpatients with cirrhosis (Fig 1). Patients had a median age of 59.4 years (IQR 53.5–65.4) and were predominantly male (65.9%) and white (79.7%) (Table 2). Most patients had cirrhosis from alcohol (30.9%) and the median admission MELD-Na score was 16.7 (IQR 14.3–22.1). Most primary admission diagnoses were liver-related (n = 74, 34.1%) or for digestive disorders (n = 64, 29.5%). Median length of stay was 3.6 days (IQR 2.4–5.7), there were 11 (5.1%) ICU transfers, and no in-hospital deaths occurred (Table 3).

**Table 2:** Prevalence of inpatient opioid use by baseline characteristics.

Characteristic		Inpatient Opioids (n = 118)	No Inpatient Opioids (n = 99)	p-value
Age (years)		58.0 (52.8–64.2)	62.6 (54.0–67.6)	**0.002**
Sex	Female	43 (58.1%)	31 (41.9%)	0.474
	Male	75 (52.4%)	68 (47.6%)	
Race	White	96 (55.5%)	77 (44.5%)	0.394
	Black	16 (57.1%)	12 (42.9%)	
	Other	6 (37.5%)	10 (62.5%)	
Body Mass Index (at admission)		28.4 (24.0–34.8)	28.1 (24.4–32.5)	0.709
Etiology of Cirrhosis	HCV	19 (63.3%)	11 (36.7%)	0.098
	Alcohol	30 (44.8%)	37 (55.2%)	
	HCV/Alcohol	23 (67.6%)	11 (32.4%)	
	NAFLD	21 (50.0%)	21 (50.0%)	
	HBV	1 (20.0%)	4 (80.0%)	
	Other	24 (61.5%)	15 (38.5%)	
MELD score (at admission)		13.9 (10.7–18.5)	15.0 (12.3–20.2)	0.101
MELD-Na score (at admission)		16.4 (10.7–21.4)	17.8 (14.9–22.3)	0.065
Cirrhosis Complications (pre-admission)	Hepatic encephalopathy	42 (51.2%)	40 (48.8%)	0.574
	Ascites	80 (54.1%)	68 (45.9%)	>0.999
	HCC	9 (52.9%)	8 (47.1%)	>0.999
	Hepatorenal syndrome	2 (28.6%)	5 (71.4%)	0.251
	Spontaneous bacterial peritonitis	10 (50.0%)	10 (50.0%)	0.815
	Esophageal varices with bleeding	80 (56.7%)	61 (43.3%)	0.318
ESRD/hemodialysis		2 (40.0%)	3 (60.0%)	0.662
Charlson Comorbidity Score		8.0 (6.0–11.0)	8.0 (6.0–11.0)	0.930
Depression		57 (67.1%)	28 (32.9%)	**0.003**
Anxiety		31 (58.5%)	22 (41.5%)	0.527
Substance Abuse/Dependence		1 (100.0%)	0 (0.0%)	>0.999
Outpatient Opioid Prescription		65 (72.2%)	25 (27.8%)	**<0.001**

Medians (IQRs) listed for all categorical variables; All statistically significant associations are in bold; HCV: hepatitis C virus; NAFLD: non-alcoholic fatty liver disease; HBV: hepatitis B virus; MELD: Model for End-Stage Liver Disease; HCC: hepatocellular carcinoma; ESRD: end-stage renal disease

**Table 3:** Hospitalization characteristics.

Characteristic		All Patients (n = 217)	Inpatient Opioid Prescription (n = 118)	No Inpatient Opioid Prescription (n = 99)	p-value
Admission Team	Family Medicine	31 (14.3%)	16 (14.9%)	15 (15.2%)	0.803
	Internal Medicine (teaching)	156 (71.9%)	87 (81.1%)	69 (69.7%)	
	Internal Medicine (non-teaching)	30 (13.8%)	15 (14.0%)	15 (15.2%)	
Non-opioid analgesics during hospitalization	NSAID	5 (2.3%)	2 (1.7%)	3 (3.0%)	0.662
	Acetaminophen	56 (25.8%)	32 (27.1%)	24 (24.2%)	0.644
	Gabapentinoid	28 (12.9%)	21 (17.8%)	7 (7.1%)	**0.024**
	TCA	4 (1.8%)	4 (3.4%)	0 (0.0%)	0.127
	SNRI	2 (0.9%)	2 (1.7%)	0 (0.0%)	0.502
	Muscle relaxant	5 (2.3%)	4 (3.4%)	1 (1.0%)	0.379
	Topical analgesic	16 (7.4%)	13 (11.0%)	3 (3.0%)	**0.035**
Benzodiazepine or hypnotic sleep aid during hospitalization		62 (28.6%)	34 (28.8%)	28 (28.3%)	>0.999
Length of stay, median (days)		3.6 (IQR 2.4–5.7)	3.9 (IQR 2.7–5.8)	3.0 (IQR 2.0–4.7)	**0.002**
ICU transfer		11 (5.1%)	5 (4.2%)	6 (6.1%)	0.554
In-hospital mortality		0 (0.0%)	0 (0.0%)	0 (0.0%)	>0.999
30-day readmission		52 (24.0%)	31 (26.3%)	21 (21.2%)	0.290

All statistically significant associations are in bold; NSAID: nonsteroidal anti-inflammatory drug; TCA: tricyclic antidepressant; SNRI: serotonin and norepinephrine reuptake inhibitor; ICU: intensive care unit

### Characteristics of inpatient opioid recipients vs non-recipients

During hospitalization, 118 patients with cirrhosis (54.4%) received opioids (Table 1). In addition, 10 patients received opioids for procedural sedation exclusively and were therefore not considered to have received inpatient opioids. In bivariable analyses, inpatient opioid administration was associated with outpatient opioid prescriptions (72.2% vs 27.8%, p<0.001), younger age (58.0 vs 62.6 years, p = 0.002), and depression (67.1% vs. 32.9%, p = 0.003). The admission diagnoses for patients receiving opioids were less likely to be infectious (4.2% vs 15.2%, p = 0.008) or mental health/substance-related (1.7% vs 12.1%, p = 0.002). Among opioid recipients, 16 (13.6%) had a primary admission diagnosis of hepatic encephalopathy.

In our multivariable logistic regression model (Table 4), younger age (OR 0.96, 95% CI 0.93–0.99) and outpatient opioid prescription (OR 3.96, 95% CI 2.13–7.56) were significantly associated with increased odds of receiving opioids during hospitalization.

**Table 4:** Adjusted multivariable analysis of opioid use.

Characteristic	OR (95% CI)
**Age (years)**	**0.96 (0.93, 0.99)**
Gender (Female = referent)	
Male	0.67 (0.35, 1.26)
Race (Black = referent)	
White	0.79 (0.31, 1.96)
Other	0.25 (0.06, 1.00)
**Outpatient Opioid Prescription**	**3.96 (2.13, 7.56)**
MELD score at admission	0.97 (0.91, 1.02)
Depression	1.89 (1.00, 3.61)
CCI	0.97 (0.89, 1.06)

Logistic regression used to calculate CI and p-values for opioid use during hospitalization; significant associations in bold; MELD: Model for End-Stage Liver Disease; CCI: Charlson Comorbidity Index

### Characteristics of opioid and non-opioid analgesic use

**Fig 2:**
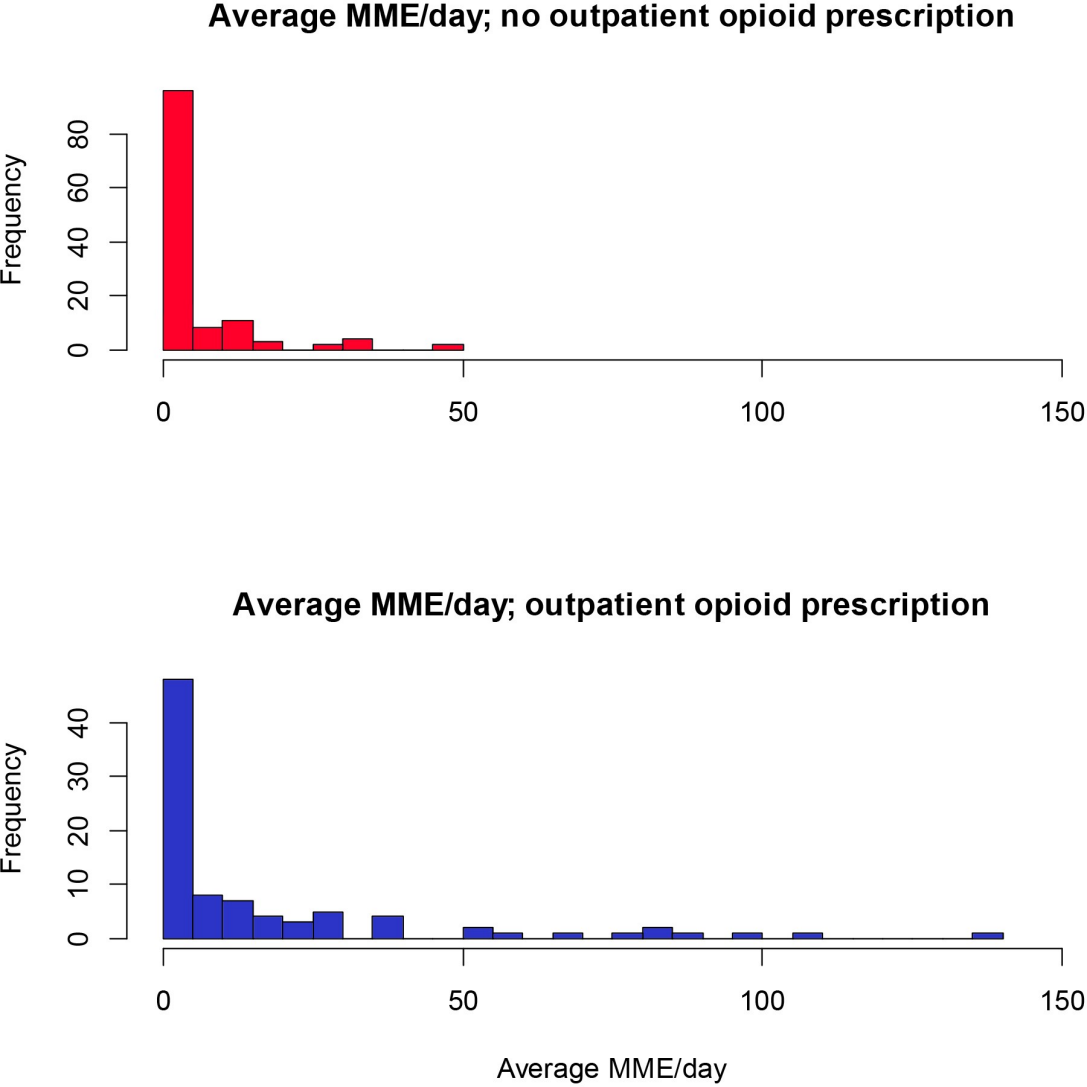
Histogram of average daily opioid use stratified by outpatient prescription of opioids. Histogram demonstrating the frequency of patients by the average morphine milligram equivalent (MME) per day among patients with and without an outpatient opioid prescription. The numerator is the total summed dose, converted into morphine equivalents, divided by the total length of hospitalization; patients on patient controlled analgesia were considered in the highest quartile.

Among all patients who received inpatient opioids (n = 118), the median daily MME was 9.8 mg (IQR 3.2–23.0) (Table 5). Opioid recipients were prescribed at least one dose for a median of 3 hospital days and 36 (30.5%) patients received opioids over ≤1 hospital day. The majority of patients received ≤ 50 MME/day, although daily doses of 50–100 MME were more common among patients with prior outpatient opioid prescriptions (Fig 2). Of opioid recipients, the most commonly prescribed opioid was oxycodone (62.7%) and 65 (55.1%) patients received multiple opioids. Benzodiazepines or hypnotic sleep aids were given to 62 (28.6%) of 276 patients overall, with no statistically significant difference between opioid recipients (28.8%) and non-recipients (28.3%) (p>0.999). The most common indication for inpatient opioids was GI-related pain (36.4%) followed by musculoskeletal (MSK) indications (33.9%) (Table 6). There was no indication for opioids mentioned within any notes for 21 (17.8%) patients.

**Table 5:** Characteristics of opioid use during hospitalization.

		Opioid Recipients (n = 118)
Median MME (mg/day), For patients with inpatient opioid prescriptions		9.8 (IQR 3.2–23.0)
Type of Medication	Codeine	0 (0.0%)
	Fentanyl*	2 (1.7%)
	Hydrocodone	1 (0.8%)
	Hydromorphone	21 (17.8%)
	Meperidine	0 (0.0%)
	Methadone	1 (0.8%)
	Morphine	43 (36.4%)
	Oxycodone	74 (62.7%)
	Propoxyphene	0 (0.0%)
	Tramadol	42 (35.6%)
	Multiple	65 (55.1%)
Opioid prescription at discharge		101 (85.6%)
New opioid prescription at discharge		22 (18.6%)

Average morphine equivalent calculated by summing each inpatient opioid administration aggregated by patient divided by individual hospitalization length in days

* Fentanyl count only includes transdermal patch and PCA

**Table 6:** Indications for opioid use during hospitalization.

Category	Cause	Count
GI-related (n = 43)	Abdominal pain (unclear etiology)	11
	SBP	5
	Ascites	7
	Cholecystitis/cholangitis/ choledocolithiasis	4
	Pancreatitis	3
	Infectious colitis/enteritis	3
	Post-procedure (e.g. variceal banding)	3
	HCC	2
	Umbilical Hernia	2
	Ileus	2
	Gastric outlet obstruction	1
Musculoskeletal (n = 40)	Chronic back pain	15
	Chronic joint pain	10
	Cellulitis	5
	Chronic ulcer	2
	Acute trauma without fracture	2
	Bone fracture	1
	Lytic bone lesions	1
	Pleurisy	1
	Pyoderma gangrenosum	1
	Leg pain from peripheral vascular disease	1
	Chest pain (presumed MSK)	1
Other (n = 12)	Headache	3
	Venous thrombosis	1
	Sickle cell anemia	1
	Laryngeal cancer	1
	Femoral neuropathy	1
	Nephrolithiasis	1
	Previously placed peritoneal catheter	1
	Generalized pain	1
	Allodynia (unclear etiology)	1
Multiple causes listed (n = 2)	Abdominal and back pain	1
	Abdominal and leg pain	1
No indication listed (n = 21)	-	-

GI: gastrointestinal; SBP: spontaneous bacterial peritonitis; HCC: hepatocellular carcinoma; MSK: musculoskeletal

At discharge, 101 patients received an outpatient prescription for opioids, 22 of which were new opioid prescriptions. Of the 22 patients with new opioid prescriptions, 19 (86.4%) had a potentially painful condition mentioned in the discharge summary including infections (cellulitis), trauma, and malignancy (HCC) (Table 7). However, others were prescribed opioids despite relative contraindications to their use (e.g. abdominal pain from ileus, C difficile colitis). Explicit documentation of the indication for opioids was provided in only 8 of 22 (36.4%) discharge summaries. The median daily MME of these opioid prescriptions was 37.5 (IQR 20.0–45.0) mg/day. Post-discharge, 14 (63.6%) of 22 patients with new opioid prescriptions at discharge received an additional opioid prescription within 30 days and 10 (45.5%) had an opioid prescription at 90 days post-discharge.

**Table 7:** Outpatient opioid prescriptions among previously opioid-naïve patients.

Characteristic	All Patients (n = 22)
Median MME (mg/day)	37.5 (IQR 20.0–45.0)
Reason for opioid documented	8 (36.4%)
Possible indication (n = 19)	SBP (n = 3) Cellulitis (n = 3) Minor trauma (n = 2) Endoscopic intervention (n = 2) HCC (n = 1) Pyoderma gangrenosum (n = 1) Degenerative disk disease (n = 1) Pancreatitis (n = 1) Periumbilical hernia (n = 1) Abdominal pain from ileus (n = 1) C. difficile colitis (n = 1) Hypertensive headaches (n = 1) Chest tube replacement (n = 1) Pleurisy (n = 1)
Additional opioid prescription within 1 month	14 (63.6%)
Active opioid prescription 90 days post-discharge	10 (45.5%)

MME: morphine milligram equivalent; SBP: spontaneous bacterial peritonitis; HCC: hepatocellular carcinoma; MSK: musculoskeletal

There were 11 patients with pre-hospitalization opioid prescriptions who were not discharged on opioids. The reason for discontinuing opioids was hepatic encephalopathy for 6 patients, polysubstance abuse in two, and altered mental status of unclear etiology in one. For two patients, there was no clear reason for stopping opioid prescriptions listed.

During hospitalization, a minority of patients received non-opioid analgesics (Table 3). Among those who received opioids, there was a significantly higher proportion who also received gabapentanoids (17.8% vs 7.1%, p = 0.024) and topical analgesics (11.0% vs 3.0%, p = 0.035). Only 27.1% of opioid recipients received acetaminophen.

### Potential complications of opioid use

Length of hospitalization was significantly longer among patients who received inpatient opioids (median 3.9, IQR 2.7–5.8 days) compared to those who did not receive opioids (median 3.0, IQR 2.0–4.7 days) (p = 0.002) (Table 3). There were similar numbers of ICU transfers among those who received opioids (4.2%) and those who did not receive opioids (6.1%) (p = 0.554). There were no in-hospital deaths in either group. Overall, 24.0% of patients had readmissions within 30 days with no significant differences between opioid recipients (26.3%) and non-recipients (21.2%) (p = 0.290).

Both inpatient opioid use (estimate 0.418, 95% CI 0.202–0.633) and higher MELD (estimate 0.034, 95% CI 0.012–0.055) were associated with length of stay in the adjusted Poisson regression model (Table 8). In the logistic regression model, only MELD (OR 1.13, 95% CI 1.03–1.25) was independently associated with ICU transfer (Table 9).

**Table 8:** Adjusted multivariable analysis of length of stay.

Length of Stay (days)	Estimate (95% CI)
**Inpatient opioid use**	**0.418 (0.202, 0.633)**
Age (years)	0.000 (-0.012, 0.011)
**MELD**	**0.034 (0.012, 0.055)**
CCI	0.026 (-0.003, 0.055)

* Poisson regression with robust standard errors used to calculate CI and p-values for length of stay; significant associations in bold; MELD: Model for End-Stage Liver Disease; CCI: Charlson Comorbidity Index

**Table 9:** Adjusted multivariable analysis of ICU transfers.

ICU Transfer	OR (95% CI)
Inpatient opioid use	0.81 (0.22, 2.95)
Age (years)	1.01 (0.95, 1.07)
**MELD**	**1.13 (1.03, 1.25)**
CCI	0.99 (0.83, 1.18)

* Logistic regression used to calculate CI and p-values for ICU transfers during hospitalization; significant associations in bold; MELD: Model for End-Stage Liver Disease; CCI: Charlson Comorbidity Index

## Discussion

In this single-center, retrospective cohort study, we found that inpatient opioid use was common among non-surgical patients with cirrhosis and associated with prolonged length of stay. Over half (54.4%) of patients received opioids during hospitalization, including 41.7% of all patients without pre-hospitalization opioid prescriptions. A significant proportion of opioid recipients (28.8%) also received benzodiazepines or hypnotic sleep aids during hospitalization. Those who received opioids were hospitalized for nearly a day longer. A total of 22 of 127 (17.3%) patients who were previously opioid-naïve received new opioid prescriptions at the time of discharge and 10 of these patients had an opioid prescription at 90 days post-discharge.

The prevalence of inpatient opioid use in our cirrhotic population was slightly higher than has been previously reported in both non-cirrhotic and cirrhotic populations. A large retrospective cohort study reported that half of non-surgical patients hospitalized in the US receive at least 1 dose of opioids [26] and a single-center, prospective cohort study reported that 41.5% of patients with cirrhosis received opioids during hospitalization [6].

We identified a statistically significant association between opioid use and prolonged length-of-stay, even after adjusting for age, MELD, and CCI. There are several potential reasons that may explain this association. Given their known side effects and decreased clearance in cirrhosis, opioids may contribute to adverse events during hospitalization including falls, delirium, hepatic encephalopathy, and opioid overdose [3, 7]. In our cohort, there was evidence of high-risk prescribing, including opioid receipt among patients with a primary diagnosis of hepatic encephalopathy and receipt of opioids in combination with benzodiazepines, which is explicitly discouraged in current guideline [13].

In addition to increasing risk of adverse events and prolonged length of stay, inpatient opioid use can lead to chronic opioid use and dependence [11, 12]. Among patients in our cohort newly discharged on opioids, 63.2% received an additional opioid prescription within 30 days of hospitalization and 45.5% had an opioid prescription 90 days post-discharge. This demonstrates the considerable risk of chronic opioid use among patients newly discharged on opioid therapy after hospitalization. Opioid use in patients with cirrhosis has also been associated with an increased risk of hepatic encephalopathy [27, 28], health care utilization [5], hospital readmissions [6], respiratory depression or overdose [29], and decreased health-related quality of life [30].

Patients with cirrhosis often require short-term analgesia during hospitalization but the lack of painful comorbidities noted in documentation suggests that opioid use could have been minimized [1, 6]. Adjunctive non-opioid analgesics were used infrequently in this cohort. Acetaminophen, which can be safely used at moderate doses (<2 g/day) in cirrhotic patients, was only given to a quarter of patients who received inpatient opioids [3]. While topical analgesics were more frequently prescribed among those who received opioids, the proportion of opioid recipients who received them was low. Gabapentin/pregabalin was used relatively infrequently but more often in opioid recipients. Combined use of gabapentin and opioids might increase the risk of adverse events since both medications can cause somnolence [3, 31].

Although our study provides novel insights on the prevalence, patterns, and complications of inpatient opioid use, it has some potential limitations. First, our population had a lower than expected number of ICU transfers (n = 11) and in-patient deaths (n = 0), precluding assessment of the potential effect of inpatient opioids on these important outcomes. However, we were able to assess the associations between opioids and length of stay and post-hospitalization opioid prescriptions. There is a potential for residual confounding by indication in our multivariable analysis of length of stay, but we attempted to reduce this risk by excluding ICU admissions/surgeries and adjusting for cirrhosis severity and comorbidities. In addition, we assessed the reason for admission and indication for opioid prescription by performing chart review. Moreover, given our exclusion criteria, our findings should not be used to guide decisions for post-surgical patients or patients referred for palliative analgesia. However, the exclusion of these patients allowed a unique investigation of inpatients with cirrhosis admitted primarily for medical reasons. It is critical to interpret these results in the context of shifting opioid-related policies. For example, in 2016, the Centers for Disease Control and Prevention changed their guidelines around opioid prescribing. Additionally North Carolina’s STOP Act, which was enacted in 2017, was aimed at curbing unsafe opioid prescribing. Given these changes in policy and the national attention on opioid safety, it is possible that opioid prescribing practices have evolved since data collection. Future research should assess time-trends in opioid prescribing [13].

In conclusion, in this study of non-surgical inpatients with cirrhosis, opioid use was common and associated with increased length of stay. Over one-fourth of opioid recipients were also given benzodiazepines or hypnotic sleep aids. A high proportion of previously opioid naïve patients were discharged on opioids and received opioid prescriptions at 90 days post-discharge. These findings provide important information on the patterns of inpatient opioid use among patients with cirrhosis and identify high-risk prescribing practices that could be addressed with quality improvement efforts.

## Supporting information

S1 File Supporting data file including all hospitalized patients with cirrhosis in this study. (XLSM)

## Abbreviations

BMI: body mass index
CCI: Charlson Comorbidity Index
CDW-H: Carolina Data Warehouse for Health
HBV: hepatitis B virus
HCC: hepatocellular carcinoma
HCV: hepatitis C virus
ICD-9-CM: International Classification of Diseases, 9th revision, clinical modification
ICU: intensive care unit
IQR: interquartile range
IRB: institutional review board
MELD: model for end stage liver disease
MME: morphine milligram equivalent
NAFLD: non-alcoholic fatty liver disease
NSAIDs: nonsteroidal anti-inflammatory drugs
SBP: spontaneous bacterial peritonitis
SNRIs: serotonin and norepinephrine reuptake inhibitors
TCAs: tricyclic antidepressants
UNC: University of North Carolina
